# New directions for emerging therapies in acute myeloid leukemia: the next chapter

**DOI:** 10.1038/s41408-020-00376-1

**Published:** 2020-10-30

**Authors:** Naval Daver, Andrew H. Wei, Daniel A. Pollyea, Amir T. Fathi, Paresh Vyas, Courtney D. DiNardo

**Affiliations:** 1 MD Anderson Cancer Center, Houston, TX USA; 2 The Alfred Hospital and Monash University, Melbourne, VIC Australia; 3 University of Colorado Department of Medicine, Division of Hematology, Aurora, CO USA; 4 Massachusetts General Hospital, Boston, MA USA; 5 MRC Molecular Haematology Unit, Weatherall Institute of Molecular Medicine, University of Oxford, Oxford Comprehensive BRC, Oxford University Hospitals NHS Foundation Trust, Oxford, UK

**Keywords:** Drug development, Acute myeloid leukaemia

## Abstract

Conventional therapy for acute myeloid leukemia is composed of remission induction with cytarabine- and anthracycline-containing regimens, followed by consolidation therapy, including allogeneic stem cell transplantation, to prolong remission. In recent years, there has been a significant shift toward the use of novel and effective, target-directed therapies, including inhibitors of mutant FMS-like tyrosine kinase 3 (FLT3) and isocitrate dehydrogenase (IDH), the B-cell lymphoma 2 inhibitor venetoclax, and the hedgehog pathway inhibitor glasdegib. In older patients the combination of a hypomethylating agent or low-dose cytarabine, venetoclax achieved composite response rates that approximate those seen with standard induction regimens in similar populations, but with potentially less toxicity and early mortality. Preclinical data suggest synergy between venetoclax and FLT3- and IDH-targeted therapies, and doublets of venetoclax with inhibitors targeting these mutations have shown promising clinical activity in early stage trials. Triplet regimens involving the hypomethylating agent and venetoclax with FLT3 or IDH1/2 inhibitor, the TP53-modulating agent APR-246 and magrolimab, myeloid cell leukemia-1 inhibitors, or immune therapies such as CD123 antibody-drug conjugates and programmed cell death protein 1 inhibitors are currently being evaluated. It is hoped that such triplets, when applied in appropriate patient subsets, will further enhance remission rates, and more importantly remission durations and survival.

## Introduction

In 2019 in the United States alone, acute myeloid leukemia (AML) was diagnosed in 21,450 new patients and resulted in 10,920 deaths^1^. AML is a highly heterogeneous disease, presenting as either de novo or secondary disease (therapy related or post-antecedent hematologic disorder). Incidence of onset increases with age, with age also associated with a higher frequency of adverse-risk cytogenetic and molecular abnormalities^2^. The median age at AML diagnosis is 67 years, with approximately one-third diagnosed above the age of 75 years^3^. In a single-center observational study, 5-year overall survival (OS) among patients with AML aged <60 years improved with time over a 16-year period from 19% to 35%. Over the same period, the 5-year OS among patients aged ≥60 did not exceed 11% (ref. ^4^). Therefore, there remains a high unmet need to improve survival and quality of life for the majority of patients with AML.

The current treatment paradigm employs remission-inducing chemotherapy, with cytarabine and anthracycline with or without a purine analogue, such as 7 days of standard-dose cytarabine plus 3 days of anthracycline (ie, “7 + 3”), fludarabine–Ara-C–granulocyte colony-stimulating factor–idarubicin, or similar induction, followed by consolidation chemotherapy and/or allogeneic stem cell transplantation (SCT) for patients with a high risk of relapse^5^. This approach has been the mainstay of therapy for the past four decades, achieving complete remission (CR) in 60–80% of patients <60 years of age. Although effective, this approach may be poorly tolerated, with a higher risk of induction mortality in patients with comorbidities, poor performance status, and/or advanced age^5–7^. In addition, unsatisfactory response rates and survival have been reported for conventional chemotherapy in patients with adverse cytogenetic risk or high-risk molecular mutations, such as *TP53* (refs. ^8–10^). Elderly patients with high-risk features and poor performance status have induction-related mortality rates in the range of 15–30% (refs. ^11–14^). Much of this early mortality is due to infectious complications, as well as organ dysfunction exacerbated by medical comorbidities. Consequently, older patients are frequently triaged or elect to receive lower-intensity regimens, which are associated with lower rates of remission, but also less early mortality^2^. Among these low-intensity regimens, hypomethylating agent (HMA) therapy has become the de facto standard of care in the United States and many other countries. In one of the largest datasets that evaluated outcomes in patients with AML aged >65 years in the real-world setting, median OS was on the order of 7–8 months, and most patients did not complete more than four cycles of HMA monotherapy, suggesting that combination HMA approaches to enhance activity, shorten time to response, and prolong remission duration and survival, while maintaining low treatment-related early mortality, are urgently needed^15^.

In recent years, there has been an increased understanding of the pathophysiology of AML, which has facilitated the development of novel, molecularly targeted therapies and the implementation of a personalized, risk-adapted approach to treatment^16–18^ (Table 1) (ref. ^19^). Particularly, in the last 3 years, nine new drugs have received US Food and Drug Administration (FDA) approval for the treatment of AML, including the B-cell lymphoma 2 (BCL-2) inhibitor venetoclax, the isocitrate dehydrogenase (IDH) inhibitors ivosidenib and enasidenib, the FMS-like tyrosine kinase 3 (FLT3) inhibitors midostaurin and gilteritinib, the anti-CD33 monoclonal antibody gemtuzumab ozogamicin (GO), the hedgehog signaling pathway inhibitor glasdegib, a liposomal formulation of a fixed combination of daunorubicin and cytarabine (CPX-351), and the oral HMA CC-486 (refs. ^16,20–23^). Although these therapies address a number of areas of unmet need in AML, much clinical research and biomarker analysis remains to be done in order to expand and optimally implement these agents (and combinations based on these agents; Table 2 [refs. ^24,25^]) in the frontline setting among fit and induction-eligible patients. With prolonged follow-up, if the remissions remain durable at 3–5 years or beyond, it is conceivable that venetoclax plus HMA or low-dose cytarabine (LDAC) may emerge as a new therapeutic backbone to enhance the activity of molecularly targeted or immune-based therapies, with potentially lower morbidity and mortality and broader usability (including older and less-fit patients, with poor performance status and organ dysfunction) than conventional intensive chemotherapy-based options, in appropriately selected patient populations.

**Table 1:** Developments in the treatment of AML (data from DiNardo et al.^19^).

1960s	Use of chemotherapy for AML introduce
1970s	Cytarabine plus anthracycline regimens (eg, 7 + 3) standard of care
1980	In younger AML patients, ASCT demonstrates OS advantage
2000	FDA approves gemtuzumab ozogamicin for R/R AML; subsequently withdrawn (2010) due to toxicities
2012	EMA (not FDA) approves decitabine for older patients with AML
2015	EMA (not FDA) approves azacitidine for older patients with AML >30% blasts
2017–2018	FDA approves
	CPX-351 for untreated t-AML or AML-MRC
	Gemtuzumab ozogamicin ± induction for CD33+ AML
	Enasidenib for R/R *IDH2*-mut AML
	Midostaurin plus induction/consolidation chemo for newly diagnosed *FLT3*-mutant AML
	Ivosidenib for R/R *IDH1*-mutant AML
	VEN + LDAC/HMA for untreated AML (older or unfit)
	Glasdegib plus LDAC for untreated AML (older or unfit)
	Gilteritinib for R/R *FLT3*-mutant AML

7 + 3, 7 days of standard-dose cytarabine plus 3 days of anthracycline.

*AML* acute myeloid leukemia, *AML-MRC* AML with myelodysplasia-related changes, chemo chemotherapy, *ASCT* allogeneic stem cell transplantation, *EMA* European Medicines Agency, *FDA US* Food and Drug Administration, *FLT3* FMS-like tyrosine kinase 3, *HMA* hypomethylating agent, *IDH* isocitrate dehydrogenase, *LDAC* low-dose cytarabine, *OS* overall survival, *R/R* relapsed/refractory, *t-AML* treatment-related AML, *VEN* venetoclax.

**Table 2:** Combination regimens with venetoclax under investigation in AML.

Doublet Venetoclax backbone	Triplet Venetoclax + HMA backbone
HMA (eg, AZA, DEC)	FLT3 inhibitor (eg, midostaurin, gilteritinib, quizartinib)
LDAC	IDH1/2 inhibitor (eg, ivosidenib, enasidenib)
FLT3 inhibitor (eg, midostaurin, gilteritinib, quizartinib)	APR-246 (TP53 target)
IDH1/2 inhibitor (eg, ivosidenib, enasidenib)	MCL1 inhibitor (CYC065, AMG 176)
MDM2 antagonist (eg, idasanutlin)	Immune therapies (CD123 ADC, CD70 antibody, PD-1 inhibitors, TIM-3 inhibitors, CD47 antibodies)
CDK9 inhibitor^a^ (eg, alvocidib, voruciclib)	
MCL1 inhibitor (S64315, AZD5991)	

*ADC* antibody-drug conjugate, *AML* acute myeloid leukemia, *AZA* azacitidine, *CDK* cyclin-dependent kinase, *DEC* decitabine, *FLT3* FMS-like tyrosine kinase 3, *HMA* hypomethylating agent, *IDH* isocitrate dehydrogenase, *LDAC* low-dose cytarabine, *MCL1* myeloid cell leukemia-1, *MDM2* mouse double minute 2, *PD-1* programmed cell death protein 1, *TIM-3 T* cell immunoglobulin and mucin domain-containing protein 3.

^a^Data from Bogenberger et al.^24^ and Luedtke et al.^25^.

## New treatment options for older unfit patients with AML

In patients aged ≥60 years who are not candidates for intensive remission-induction therapy, venetoclax plus either HMA or LDAC have emerged as an effective treatment option on the basis of a high and rapid rate of response achievement. Recently published phase 3 studies validated a survival benefit for azacitidine plus venetoclax, compared with azacitidine plus placebo^26^. For LDAC plus venetoclax, a survival improvement was observed only in a post-hoc analysis with longer follow-up^27^. These studies have established a new path forward for standard-of-care therapy in patients unfit for intensive chemotherapy. Future questions now include: (1) which older and/or unfit patients benefit most from this low-intensity approach, rather than intensive chemotherapy (defining fitness and eligibility for induction chemotherapy or the lack thereof remains a topic of much debate and discussion in the AML field); (2) the utility of venetoclax (with HMA or with other backbones) in other AML settings, such as relapsed/refractory AML; (3) the feasibility of combining venetoclax with intensive chemotherapy in fit, younger patients; and (4) the potential for venetoclax in the maintenance phase of therapy for patients in CR.

Cytarabine plus anthracycline induction, as part of an intensive regimen, may still be appropriate for some patients ≥60 years of age, particularly those without serious comorbidities and good performance status, where remission rates of 60–70% may be achieved^7^. With the recent introduction of the oral BCL-2 inhibitor venetoclax in combination with either HMA or LDAC, combined CR/CR with incomplete blood count recovery (CRi) response rates of 54–73% have been reported in untreated older patients at selected phase 2 doses, with early mortality rates of 3–7% (refs. ^28–30^). Reflecting the dearth of effective new agents for older patients with AML, in November 2018, the FDA awarded accelerated approval to venetoclax in combination with azacitidine, decitabine, or LDAC in untreated patients with AML aged ≥75 years, or in patients with comorbidities that preclude the use of intensive induction chemotherapy^31^. The sustained future of venetoclax in previously untreated patients with AML considered ineligible for intensive chemotherapy due to age or comorbidities has been reinforced by the positive outcome from the recently completed randomized phase 3 study that evaluated azacitidine (VIALE-A) with or without venetoclax, with OS as the primary endpoint^26^. A parallel study also examined the role of LDAC with venetoclax or placebo in the VIALE-C trial. The preplanned primary OS analysis (median follow-up: 12 months) of VIALE-C failed to meet its primary endpoint, with a median OS of 7.2 months versus 4.1 months, respectively (hazard ratio [HR] 0.75 [95% CI: 0.52, 1.07]; *P* = .11) (ref. ^32^). Although the primary endpoint was not met, a 6-month update of study outcomes reported a 30% reduction in the risk of death (HR 0.70 [95% CI: 0.50, 0.99]; *P* = .04). Median OS in the 6-month update was 8.4 months in the venetoclax arm versus 4.1 months in the placebo arm^33^.

The dual primary endpoints of the VIALE-A study (OS and CR or CRi [composite CR] rate) have been met and data have recently been published. With a median follow-up of 20.5 months, venetoclax plus azacitidine demonstrated a statistically significant and clinically meaningful improvement in OS and response rates compared with placebo plus azacitidine (OS: 14.7 vs 9.6 months; HR 0.66 [95% CI: 0.52, 0.85]; *P* < .001; CR/CRi: 66% vs 28%, *P* < .001) in treatment-naive patients with AML ineligible for intensive therapy. The incidence of CR was higher with azacitidine-venetoclax than with the control regimen (36.7% versus 17.9%; *P* < .001), as was the composite CR (66.4% versus 28.3%; *P* < .001). These results confirmed the phase 1b response and OS findings and further solidified the efficacy of the azacitidine and venetoclax combination in patients with AML^26^.

The HMA plus venetoclax regimen has been approved by the FDA and predominantly used in patients who are older and considered unsuitable for induction therapy, often determined on the basis of high predicted/perceived induction mortality in the opinion of the treating physician. Some patients, however, may significantly improve their performance status and organ function after achieving remission and may become candidates for allogeneic SCT later. In an aggregated analysis of 304 patients treated with venetoclax-based therapy in two global, open-label phase 1b (NCT02203773) and phase 1/2 (NCT02287233) clinical trials studying venetoclax in combination with the HMAs decitabine or azacitidine, and LDAC, respectively, 31 patients (10%) proceeded to receive allogeneic SCT; all of these patients were treated in the US^27^. About 55–60% of patients remained in remission at 1 year post-allogeneic SCT, 40% remained in remission at 2 years post-allogeneic SCT, and 68% were alive at 2 years post-allogeneic SCT. These data suggest that allogeneic SCT after HMA-plus-venetoclax-based therapies is safe and effective, offers potential for long-term remissions, and could be considered in appropriate patients.

Another major remaining question is the optimal duration of therapy with HMA-plus-venetoclax-based regimens. Traditionally, therapy with HMAs has been continuous and indefinite, until progression or intolerance. Given the early, deep, and durable responses with HMA plus venetoclax, interest has emerged in potentially curtailing or discontinuing therapy in patients who have deep responses. Although this appears biologically plausible, no clinical data at this time clearly identify a population in whom HMA and venetoclax therapy can be stopped with a high degree of confidence that relapse will not occur. This is an area ripe for clinical trials to examine controlled discontinuation of one or both agents on the basis of emerging MRD-assessing technologies, including flow cytometry, next-generation sequencing, droplet digital polymerase chain reaction, and/or combinations of these modalities. Staged discontinuation of one or both agents under close monitoring could be considered, to evaluate the feasibility and safety of such an approach. Molecular subtypes known to be highly sensitive and likely to have deeper responses could also be examined, or potentially the setting of triplets that are anticipated to give deep and early responses and potentially be “curative.” At this time, however, the authors do not believe HMA or venetoclax therapy should be routinely discontinued, as long as it is tolerable and maintained efficacy is observed.

Early clues to molecular biomarkers of response and resistance to venetoclax were derived from the clinical experience in relapsed/refractory patients with AML, where venetoclax monotherapy resulted in a response rate of 19% (ref. ^34^). Molecular correlates of sensitivity were *SRSF2/ZRSR2* and *IDH1/2* mutations, with four of twelve (33%) patients with *IDH1/2* mutations achieving a CR or CRi^34^. Conversely, *FLT3* and *PTPN11* mutations were associated with primary and secondary resistance to single-agent venetoclax, with lower response rates, shorter time on therapy^35^, and acquisition or enrichment of FLT3 among responders at the time of loss of response. Recent correlative analyses from a cohort of patients enrolled in the phase 1b/2 studies of venetoclax in combination with either HMA or LDAC provide further insights into molecularly based outcomes in previously untreated older AML patient populations^36^. In terms of durable responses and high rates of 1+ year survival, the associations with *NPM1* and *IDH2* mutations were most notable, with *NPM1*-mutant clones durably erased for >3 years in the majority of patients examined. In contrast, among patients with adaptive resistance, enrichment or acquisition of clones dominated by *FLT3*-ITD or biallelic *TP53* defects were observed, highlighting the importance of screening and identifying these lesions at the time of treatment failure. In the recently published VIALE-A phase 3 study, the molecular biomarkers of response and survival were confirmed, with encouraging CR/CRi rates >75% with azacitidine and venetoclax in *IDH1* or *-2*–mutated AML compared with azacitidine alone^26^. *FLT3*-mutated patients continued to show high CR/CRi rates >65%, but data on stratification by *FLT3*-ITD versus -TKD, *FLT3* allelic ratio, and *NPM1* co-mutation status, factors that significantly impact the biologic and clinical outcomes in *FLT3*-mutated AML, were not presented and are eagerly awaited. *TP53* mutations continue to be associated with poor efficacy to HMA plus venetoclax, with CR/CRi rates of ~50% and median OS of 5–7 months across numerous studies. Furthermore, early identification of these lesions may enable the concomitant or sequential implementation of therapies to target *FLT3* or those being developed for *TP53*, with the goal of enhancing the rate and duration of responses.

Glasdegib, an oral small-molecule inhibitor of the Smoothened protein and the Hedgehog signaling pathway, in combination with LDAC was FDA approved in 2018, for patients with AML or high-risk myelodysplastic syndrome (MDS) unsuitable for intensive chemotherapy. The BRIGHT AML 1003 study (NCT01546038) randomized 132 older or unfit AML patients to receive the combination (*n* = 88) versus LDAC alone (*n* = 44) (ref. ^37^). The OS was superior in those receiving LDAC with glasdegib versus LDAC monotherapy, with a median OS of 8.3 months for the combination, versus 4.3 months for monotherapy (*P* = .0002). The rate of CR was also superior among those receiving the combination (18.2% for the combination versus 2.6% for monotherapy). Despite the positive readout, the efficacy of glasdegib plus LDAC appears modest when compared with the data of HMA-venetoclax combinations in similar, unsuitable for intensive chemotherapy patient populations, albeit with the caveat and hazards of comparisons across distinct clinical trials. For this reason, azacitidine plus venetoclax appears to have become the favored approach for frontline treatment of AML in patients unsuitable for intensive therapy.

Another subgroup of high-risk AML patients are those with secondary AML, including AML from an antecedent hematologic disorder, AML with myelodysplasia-related changes (AML-MRC) as defined by the World Health Organization, and therapy-related AML (ie, AML in patients who have received chemo- and/or radiation therapy for prior conditions). CPX-351, a liposomal dual-drug encapsulation of cytarabine and daunorubicin, appeared to be particularly effective in patients with secondary, therapy-related AML (t-AML), or AML-MRC. In a phase 3 randomized study in patients 60–75 years old with t-AML, secondary AML, or AML-MRC, CPX-351 demonstrated a significantly higher rate of CR (47.7% versus 33.3%; *P* = .016) and superior median OS (9.56 versus 5.95 months; HR 0.69 [95% CI: 0.52, 0.90]; *P* = .003) compared with the existing standard of conventional 7 + 3 induction^38^. CPX-351 appeared to be well tolerated, with reduced 30- and 60-day mortality, more patients transitioning to allogeneic SCT, and improved post-transplant survival, as well as recently updated improved 5-year OS compared with 7 + 3 in the target population^39^. These data led to the FDA approval of CPX-351 as induction therapy in patients with secondary AML, t-AML, or AML-MRC. It must be noted that CPX-351, while better-tolerated and more effective than 7 + 3 in this population, is still a combination of anthracycline and cytarabine, and should be viewed as an intensive induction approach, and not a lower-intensity alternative to be considered in patients who are deemed unsuitable for intensive therapy.

Recent data showed that CC-486, an oral HMA, significantly improved OS when administered as a maintenance therapy after 3 + 7-based induction and consolidation therapy in patients 55 years of age or older who did not proceed to allogeneic SCT. These data resulted in approval of CC-486 as the first oral HMA to be approved in AML, on September 3, 2020. Although the initial approval and use of CC-486 is expected to be in the maintenance setting, on the basis of the phase 3 target population of the registration study, a number of trials evaluating CC-486 in the newly diagnosed and relapsed/refractory AML settings in combination with venetoclax and targeted/immune therapies are anticipated or ongoing. The availability of this drug also opens the possibility and the hope of a completely oral regimen for AML in the future, if combination trials of CC-486 with venetoclax or targeted agents show similar efficacy to that seen with the intravenous azacitidine formulation in similar combinations. However, this needs to be evaluated in clinical trials to confirm efficacy and safety before standard usage, but is an exciting potential possibility for the future.

## Opportunities to expand the therapeutic landscape

The deep and durable remissions achieved in some older patients using venetoclax and azacitidine may at least partially be explained by the disruption of energy metabolism that occurs in leukemia stem cells (LSCs) in response to this drug combination. LSCs, in contrast to normal hematopoietic cells, are characterized by their dependence on amino acid metabolism^40^ and oxidative phosphorylation^41^. Venetoclax with azacitidine reduces the uptake of amino acids into LSCs^40^, resulting in decreased oxidative phosphorylation and enhanced vulnerability of the LSC population, whose metabolic inflexibility prevents switching to another form of metabolism for survival^42^. LSCs from patients with relapsed AML, or those refractory to venetoclax with azacitidine, are able to compensate for reduced amino acid metabolism and may be one of the potential reasons for lower response rates, shorter remission durations, and shorter OS noted with venetoclax and azacitidine/decitabine in patients with relapsed/refractory AML^43^.

A recent analysis by Pei et al showed that the stage of differentiation of leukemia could be a powerful predictor of nonresponse to the combination of azacitidine/decitabine with venetoclax^43^. Specifically using FAB AML M5 as a surrogate, monocytic morphology was an independent predictor of resistance in 100 newly diagnosed patients uniformly treated with HMA/venetoclax. This was likely due to the downregulation of BCL-2 and upregulation of myeloid cell leukemia-1 (MCL1) that is a conserved feature of normal hematopoiesis (human and mouse) as cells differentiate. The authors further demonstrated that MCL1 takes over as the driver for oxidative phosphorylation in monocytic LSCs, driving resistance to venetoclax-based therapies.

## Could biologically directed therapies deprioritize traditional age-based barriers?

**Fig. 1:**
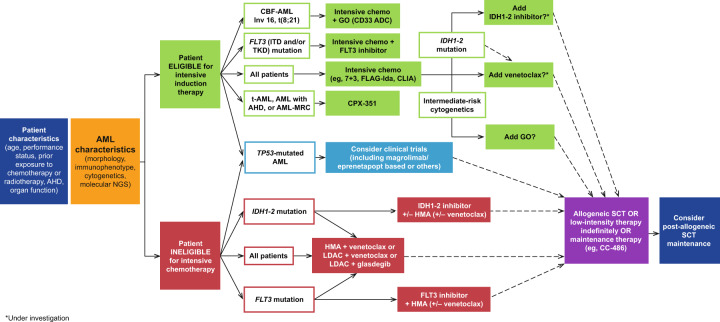
Newly diagnosed AML: advancements in the diagnostic and treatment paradigm. 7 + 3, 7 days of standard-dose cytarabine plus 3 days of anthracycline; ADC antibody-drug conjugate; AHD antecedent hematologic disorder; AML acute myeloid leukemia; AML-MRC AML with myelodysplasia-related changes; CBF core binding factor; CLIA cladribine–idarubicin–Ara-C; CPX-351 liposomal formulation of a fixed combination of daunorubicin and cytarabine; GO gemtuzumab ozogamicin; FLAG-Ida fludarabine–Ara-C–filgrastim plus idarubicin; FLT3 FMS-like tyrosine kinase; HMA hypomethylating agent; IDH isocitrate dehydrogenase; LDAC low-dose cytarabine; NGS next-generation sequencing; SCT stem cell transplantation; t-AML therapy-related AML.

The availability of less-intensive treatment options with encouraging efficacy in patients aged >60 years may lead to a reduced emphasis on patient-related factors, such as age and comorbidities, as treatment considerations, and instead increase the impetus to tailor treatments on the basis of the molecular and cytogenetic profile (Fig. 1). Determining an individual’s underlying genomic status is becoming increasingly routine as part of initial diagnostic procedures for AML. This knowledge has important immediate implications for clinical decision-making related to therapy (eg, donor selection and conditioning regimen for transplantation), detection of an underlying genetic predisposition, identification of novel AML subclasses, and prognosis^44,45^. Perhaps most importantly, knowledge of a patient’s genomic status may be relevant in selecting an optimal induction regimen, such as all-trans retinoic acid plus arsenic trioxide for acute promyelocytic leukemia^46^, adding GO in core binding factor AML^47^, CPX-351 in patients with myelodysplasia-related changes of therapy-related AML^48^, adding midostaurin for *FLT3*-mutated AML^47^, or considering IDH inhibitor-based approaches for *IDH*-mutant AML.

In the current AML setting, expeditious confirmation of *IDH1/2* and *FLT3* mutation status is particularly important, together with p53, core-binding factor, and myelodysplasia-related changes-associated cytogenetics, given the availability of targeted therapies that improve outcomes in patients with relevant molecular or cytogenetic features^3^. In the case of *IDH*- and *FLT3*-mutated AML, a number of molecularly targeted combination regimens are emerging as alternatives to or building on the 7 + 3 regimen. These include azacitidine plus an IDH inhibitor for *IDH*-mutated AML in older or less-fit patients, midostaurin plus 7 + 3 in fit *FLT3*-mutated AML, or consideration of FLT3 inhibitors in combination with azacitidine in older or less-fit *FLT3*-mutated AML patients^3^. In terms of the time required to identify a targetable mutation in a newly diagnosed patient with AML, retrospective analyses suggest that patient outcomes are not compromised by a short delay from the time of diagnosis to commencement of induction treatment, both in younger and older patients, when monitored closely^49^. Thus, a valid approach would be to first identify patients with nonproliferative, stable disease able to wait for 5–10 days with close monitoring (the overwhelming majority of new AML patients), to enable identification and selection of an optimal induction regimen^31,49^.

## The evolving role of hybrid regimens co-targeting BCL-2 and molecularly actionable AML subgroups

Venetoclax is currently most extensively used in combination with HMA or LDAC, but in patients with targetable mutations it is foreseeable that therapy may be optimized with the substitution of azacitidine for a molecularly targeted therapy (molecular-targeted doublet) or the addition of a molecular-targeted therapy to a venetoclax-HMA backbone (triplet) with appropriate dose adjustments, interruptions, and use of growth factors for potential cumulative myelosuppression. In addition, triplet regimens may have the potential to enhance therapeutic value in selected patient populations harboring targetable mutations, such as *IDH1/2* and *FLT3*, or antigen targets such as CD33, CD123, C-type lectin domain family 12 member A, and programmed cell death protein 1 (PD-1). This approach has parallels with multiple myeloma, where the decades-long traditional conventional induction for newly diagnosed patients with vincristine, anthracycline, and dexamethasone was replaced by combinations of immunomodulatory drugs, proteasome inhibitors, and plasma cell-targeted monoclonal antibodies, on the basis of improved efficacy and safety. At the same time, it is important to note that there remain certain favorable European LeukemiaNet disease subsets, such as core-binding factor AML, *NPM1*-mutated AML, and *FLT3* wildtype AML, wherein finite duration of induction and consolidation therapy such as 3 + 7 with or without GO, fludarabine–Ara-C–filgrastim (FLAG), or FLAG-GO (±idarubicin) may be curative, with long-term remissions in >70–75% of patients^50–53^. We believe that such patients are likely to be most effectively treated in the current landscape with traditional anthracycline-cytarabine–based therapies with a curative intent, and may not be ideal patients to consider for HMA-plus-venetoclax-based therapies, at least until long-term follow-up and more-mature data and/or trials in these specific subsets provide evidence to the contrary.

### FLT3 mutations

Mutations in *FLT3* (including ITD and TKD) are present in around one-third of patients with AML. *FLT3-*ITD mutations with high allele ratio (>0.5) have historically been associated with inferior prognosis^54,55^. Several FLT3 inhibitors have been evaluated in clinical trials of patients with AML^56^. Among these, the second-generation FLT3 inhibitors quizartinib and gilteritinib as single agents improved marrow remission rates and OS compared with traditional salvage chemotherapies (investigator’s choice of high-intensity therapies such as fludarabine–Ara-C–granulocyte colony-stimulating factor–idarubicin or mitoxantrone-etoposide-cytarabine, or low-intensity therapies such as HMAs or LDAC) in relapsed/refractory *FLT3*-mutated AML patients^57–60^. Midostaurin is used in combination with induction chemotherapy for the first-line treatment of patients with *FLT3-*ITD– or *FLT3-*TKD–mutated AML^61^. To date, midostaurin and gilteritinib have been approved by the FDA for the treatment of *FLT3*-mutated AML, with multiple other FLT3 inhibitors in advanced clinical development, including quizartinib and crenolanib^56,60^. Further clinical trial data supporting the combination of chemotherapy and other FLT3 inhibitors are awaited.

### IDH1/2 mutations

*IDH1* and *IDH2* mutations result in the aberrant production of 2-hydroxyglutarate, which leads to DNA and histone hypermethylation and impaired myeloid differentiation, promoting oncogenesis in AML^62,63^. *IDH1/2* mutations are found in around 20% of patients with AML^64^. Among the IDH inhibitors that have been evaluated in clinical trials of patients with AML, ivosidenib and enasidenib have been approved by the FDA for the treatment of *IDH1*- and *IDH2*-mutated disease, respectively^65^. In a phase 1 study of patients with *IDH1*-mutated relapsed/refractory AML, ivosidenib monotherapy achieved a CR/CRi rate of 30.4% with a median duration of response of 8.2 months^66^. Similarly, a phase 1/2 study of single-agent enasidenib in patients with *IDH2*-mutated relapsed/refractory AML reported a CR rate of 19.6%, with similar overall response rates (ORR) among patients in relapse (38%), or who were refractory to intensive (38%) or nonintensive (43%) therapies^63^. Another phase 1/2 study evaluating enasidenib as a single agent in older patients with newly diagnosed *IDH2*-mutated AML reported an ORR of 31%, including CR in 18% (ref. ^67^). A phase 1/2 study evaluating single-agent ivosidenib in frontline older and unfit patients with newly diagnosed *IDH1*-mutated AML reported an ORR of 42% and CR rate of 30% (ref. ^68^).

Although results have not yet been published, a phase 3 trial comparing the IDH2 inhibitor enasidenib with a variety of conventional care regimens in patients with *IDH2*-mutant AML after failure of two or three lines of prior therapy did not meet its primary endpoint of OS^69^.

IDH inhibitors combine synergistically with azacitidine, with preliminary findings of phase 2 studies supporting a potential role for azacitidine combinations with either enasidenib^18^ or ivosidenib^70^ in patients with newly diagnosed AML and *IDH2* or *IDH1* mutations, respectively. Similarly, FLT3 inhibitors have shown clinical synergy in both the frontline and relapsed setting when combined with HMAs or LDAC, although long-term survival remains poor^71–73^.

Several clinical studies are ongoing with FLT3 and IDH1/2 inhibitors in combination with venetoclax. Among these, a phase 1b/2 study is currently evaluating venetoclax and quizartinib in patients with *FLT3*-mutated relapsed/refractory AML (NCT03735875). Preclinical data show that venetoclax is highly synergistic when combined with the FLT3 inhibitors gilteritinib^74^ and quizartinib^35^. Combination treatment demonstrated enhanced apoptosis resulting from venetoclax mitigating the unintended prosurvival effects of the tyrosine kinase inhibitor and possibly from MCL1 inhibition by the FLT3 inhibitor, thereby abrogating venetoclax resistance via alternate proapoptotic pathway upregulation^74^. A subsequent phase 1b study (NCT03625505) in patients with wildtype or *FLT3*-mutated relapsed/refractory AML showed that the combination of venetoclax and gilteritinib was highly active in patients with *FLT3*-mutated disease, 60% of whom had received one or more prior FLT3 tyrosine kinase inhibitor-based therapies^75^. Fifty percent of patients with mutant *FLT3* achieved composite CR (CR plus CRi plus CR with incomplete platelet recovery) with an ORR (CR plus CRi plus morphologic leukemia-free state) of 88% in *FLT3*-mutated patients. The ORR rates were maintained in patients with prior FLT3 inhibitor exposure and in ITD/TKD populations. This compares favorably with ORRs of 35–55% noted in phase 1/2 and 3 studies with quizartinib^57,76^ and gilteritinib^58,77^, in patients with relapsed/refractory *FLT3-*mutated AML – even though the patients in these trials were generally more favorable, with only 4–11% of patients exposed to prior tyrosine kinase inhibitor-based therapy.

*IDH1*- and *IDH2*-mutant primary AML cells are more sensitive to venetoclax compared with wildtype *IDH1/2* cells^78^, with durable responses and encouraging OS seen in *IDH*-mutated patients treated with venetoclax regimens. A phase 1b/2 study of venetoclax in combination with enasidenib in *IDH2*-mutated AML is currently ongoing (NCT04092179). A separate phase 1b/2 study investigating the combination of venetoclax and ivosidenib (with or without the incorporation of azacitidine as the “triplet”) for patients with *IDH1*-mutated myeloid diseases (relapsed/refractory or treatment naive ineligible for standard induction chemotherapy) has enrolled 19 patients and reported a CR/CRi in 78% of patients^79^, with 50% of responding patients achieving minimal residual disease-negative status by flow cytometry.

A number of novel strategies are also being investigated for the treatment of patients without targetable mutations, with various potential venetoclax doublet regimens that are based on preclinical synergy/rationales currently under investigation. These include the mouse double minute 2 (MDM2) antagonist idasanutlin, MCL1 inhibitors, mitogen-activated protein kinase inhibitors, PD-1 inhibitors, and antibody-drug conjugates (ADCs). The rationale for using MDM2 inhibitors as a potential combination partner for venetoclax relates to their TP53-modulating ability, with enhanced wildtype *TP53* function, downstream inhibition of MCL1, and activation of proapoptotic pathways (PUMA, BAX, BIM) achieved with MDM2 inhibition^80^. A phase 1b study in patients aged ≥60 years with relapsed/refractory AML or previously rated secondary AML reported an antileukemic response rate (CR plus CR with incomplete platelet recovery plus CRi plus morphologic leukemia-free state) for the combination of venetoclax plus idasanutlin of 41% across all dose levels, with an antileukemic response rate of 50% for the two venetoclax 600-mg cohorts being considered for the recommended phase 2 expansion^81^. Minimal residual disease negativity was achieved in five of 11 (45%) patients with CR, CR with incomplete platelet recovery, or CRi.

Preclinical rationale has also been established for combining venetoclax with either an MCL1 or mitogen-activated protein kinase inhibitor. MCL1 inhibition has been shown to rapidly induce a committed step toward apoptosis in tumor cell lines. The combination of MCL1 inhibition and BCL-2 mimetics concomitantly target both antiapoptotic pathways BCL-2 and MCL1 with highly synergistic effects in AML models and patient samples^82–84^. The MCL1 inhibitors S64315 (NCT03672695), CYC065 (NCT04017546), AMG 176 (NCT03797261), and AZD5991 (NCT03218683) are currently being evaluated in phase 1 or phase 1/2 clinical trials in combination with venetoclax in patients with AML.

Immune checkpoint inhibition is an effective treatment strategy in multiple solid tumors and is emerging as a potential strategy in hematologic malignancies. Preclinical studies have now shown that venetoclax treatment selectively spares activated (central and effector memory T cells) but not naive T-cell and B-cell populations. Venetoclax did not antagonize, and in some tumor models enhanced the therapeutic effects of anti–PD-1 treatment^85^. Moreover, the resistance of natural killer cells to venetoclax-induced cell death in mouse models suggests that venetoclax may favorably skew the immune response, supporting its combination with PD-1 and PD-1 ligand 1 (PD-L1) inhibitors^86^.

The safety and efficacy of azacitidine in combination with PD-1 or PD-L1 inhibitors in AML has been evaluated in recent studies. An open-label phase 2 trial of azacitidine in combination with nivolumab in patients with relapsed/refractory AML (*n* = 70) reported that the combination was safe and effective, with encouraging response rates and OS specifically in HMA-naive and salvage 1 patients with relapsed AML^87^. The ORR was 33%, including 15 (22%) CR/CRi (CR: *n* = 4; CRi: *n* = 11), one partial response (PR), and seven patients with hematologic improvement maintained >6 months. In addition, six (9%) patients remained on study with stable disease (SD) > 6 months. In HMA-naive (*n* = 25) and HMA pretreated (*n* = 45) patients, the ORR was 58% and 22%, respectively^87^. In-depth biomarker analysis using mass cytometry and flow cytometry demonstrated that patients who had higher pretherapy bone marrow CD8 or CD3 infiltration had significantly higher response rates. This suggests that similar to molecular mutation heterogeneity, there may be significant immune heterogeneity in AML, and that patient selection using immune biomarkers may help identify patients most likely to benefit from this regimen, thereby optimizing the risk-benefit ratio with such immune therapies in AML. Another phase 2 study evaluated azacitidine plus pembrolizumab in patients with relapsed/refractory AML and in older (≥65 years) patients with newly diagnosed AML. The combination was well tolerated in both patient populations. Among evaluable patients with relapsed/refractory AML (*n* = 29), four achieved CR/CRi (CR: *n* = 2; CRi: *n* = 2); one (4%) PR, four (14%) hematologic improvement, and seven (24%) SD (six or more cycles) were also reported. With a median follow-up of 14.9 months, the median OS for the relapsed/refractory cohort was 10.8 months. Among evaluable patients with newly diagnosed AML (*n* = 17), eight (47%) achieved CR/CRi (CR: *n* = 6; CRi: *n* = 2); two (12%) PR, two (12%) hematologic improvement, and four (24%) SD (six or more cycles) were also reported. With a median follow-up of 19 months, the median OS for the newly diagnosed cohort was 13.1 months^88^. Finally, a randomized phase 2 study of azacitidine alone (*n* = 42) or in combination with the PD-L1 inhibitor durvalumab (*n* = 42) in older (≥65 years) patients with AML reported similar efficacy for azacitidine alone or in combination, with no new safety signals or potential overlapping risks identified with the combination regimen^89^. The ORR (CR plus CRi) of combination therapy was 31% (CR: *n* = 11; CRi: *n* = 9), and 35% (CR: *n* = 14; CRi: *n* = 9) for azacitidine alone^89^. A triplet combination of azacitidine-venetoclax with PD-1 inhibitor nivolumab to further enhance the response rates and duration of response in frontline older AML not fit for traditional induction and relapsed/refractory AML is being investigated (NCT02397720). In addition, it is important to note that one of the first studies to show efficacy with an immune checkpoint inhibitor in AML was with the cytotoxic T-lymphocyte antigen-4 inhibitor ipilimumab in patients with relapsed AML post-allogeneic SCT, with five of 12 post-allogeneic SCT patients achieving response to single-agent ipilimumab^90^. It must be noted, however, that the patients in this study had >99% donor chimerism, were ~12–16 months post-allogeneic SCT with no active and no history of grade 3/4 graft-vs-host disease, and all responses were seen with a higher-than-standard dose of ipilimumab (10 mg/kg every 3weeks). Nonetheless, these findings are striking and highlight the ability to modulate the immune system to generate antileukemic activity, and a potential role of ipilimumab either in combination with PD-1/PD-L1 inhibitors or on its own.

The combination of ADC and venetoclax is a further possibility for venetoclax doublet (and potentially triplet) regimens. Preclinical studies of IMGN632, a CD123-targeting ADC, in combination with azacitidine and/or venetoclax support the evaluation of these combinations in patients with AML. The addition of IMGN632 to azacitidine alone or in combination with venetoclax led to significantly improved survival in multiple AML xenografts and patient-derived xenograft models^91^. Further, results from a phase 1 trial of IMGN632 in patients with CD123-positive relapsed/refractory AML demonstrated a manageable safety profile and promising single-agent activity: CR/CRi 19–36% across different AML subsets, with most of the responders (92%) having experienced failure of prior intensive therapies. IMGN632-related toxicities did not lead to treatment discontinuations, and no patterns of hepatotoxicity or cytopenias occurred with doses below 0.18 mg/kg^92^. These encouraging results prompted the initiation of a phase 1b/2 study evaluating the safety and antileukemia activity of IMGN632 when administered in combination with azacitidine and/or venetoclax in patients with relapsed and previously untreated CD123-positive AML (NCT04086264).

### Venetoclax triplet therapy and future directions

As the treatment paradigm for multiple myeloma has expanded to include the combination of several agents in the induction and salvage setting to improve survival, so too there is interest in combination or sequential approaches for AML treatment. Triplet regimens of venetoclax, HMA, and FLT3 inhibitor are already being investigated (Table 2 [refs. ^24,25^]). One phase 1/2 study is currently evaluating the combination of quizartinib, decitabine, and venetoclax in patients with untreated or relapsed AML/MDS (NCT03661307); another phase 1/2 study is investigating gilteritinib together with azacitidine and venetoclax in relapsed/refractory *FLT3*-mutated AML and high-risk MDS (NCT04140487). In addition, a phase 1/2 clinical trial is investigating the IDH1 inhibitor ivosidenib and venetoclax with or without azacitidine for the treatment of patients with *IDH1*-mutated AML (NCT03471260). Similarly, a phase 1/2 clinical trial is evaluating the combination of HMA-venetoclax with the TP53-targeted therapy APR-253, which showed high efficacy in combination with azacitidine in *TP53*-mutated frontline MDS and AML. Identifying the optimal duration of therapy for venetoclax and the targeted agent deployed, selecting concomitant versus sequential administration of these agents, the ideal timepoint for bone marrow evaluation to allow for early interruption, and the timing and need for growth factor support are questions these trials will attempt to answer. It is hoped that such triplets will enhance efficacy while maintaining an acceptable safety profile and, importantly, early mortality rates <5–10%.

## Conclusions

In the past few years, there has been a rapid shift toward the use of oral small-molecule and targeted therapies in AML, including the approval by the FDA of the BCL-2 inhibitor venetoclax in combination with HMA or LDAC in elderly (≥75 years) patients or those with comorbidities that preclude intensive induction in newly diagnosed AML. As a heterogeneous disease associated with many high-risk molecular and cytogenetic features, the treatment landscape has also expanded to include novel molecular-targeted therapies for patients with mutations such as *FLT3* and *IDH1/2*, and potentially for *TP53*. With increased awareness of AML has come the realization that disease-specific factors, and not patient factors such as age and performance status, maybe the critical determinants of outcome. Consequently, there is an emerging interest for venetoclax not only in older and unfit patients, but potentially also in younger patients with adverse biologic features, although further data from ongoing and planned randomized trials in such populations are needed to support such a paradigm shift. As treatment centers move toward the use of increasingly precise and personalized treatment plans, less toxic doublet and triplet regimens in appropriate patient populations when appropriately administered as guided by clinical trials may eventually provide several advantages. These include the potential to improve quality of life, increase time outside the hospital, reduce early mortality and organ damage, and it is hoped, thereby reduce the clinical, emotional, and psychosocial burden associated with current intensive therapies. It is not yet clear whether an additional burden in AML, the financial burden, will decrease with newer therapies. Although patients may spend less time hospitalized, thereby lessening overall medical costs, receiving these novel regimens and combinations undoubtedly incurs high costs, frequently tens of thousands of dollars per month^93^. This is an important consideration, as newer and more effective regimens emerge for AML, and the question arises of how they will be incorporated into standard approaches. Nevertheless, despite looming challenges, the road ahead seems much more promising today than it was a decade ago!

## Disclaimer

AbbVie was allowed a courtesy review of the final paper.
